# Mogamulizumab Treatment in a Hemodialysis Patient with Adult T-Cell Leukemia/Lymphoma

**DOI:** 10.4274/tjh.2014.0166

**Published:** 2014-12-05

**Authors:** Mari Yoshihara, Yasushi Kubota, Makoto Fukuda, Tomoya Kishi, Yuji Ikeda, Shinya Kimura

**Affiliations:** 1 Saga University Faculty of Medicine, Department of Internal Medicine, Division of Hematology Respiratory Medicine, and Oncology, Saga, Japan; 2 Saga University Faculty of Medicine Hospital, Department of Transfusion Medicine, Saga, Japan; 3 Saga University Faculty of Medicine, Department of Internal Medicine, Division of Nephrology, Saga, Japan

**Keywords:** Adult T-cell leukemia/lymphoma, Mogamulizumab, Hemodialysis, CCR4, HTLV-1

## TO THE EDITOR

Here we describe, for the first time, a hemodialysis patient suffering from adult T-cell leukemia/lymphoma (ATL) who was treated with mogamulizumab, a defucosylated anti-CC chemokine receptor 4 (CCR4) monoclonal antibody [1].

An 83-year-old female was admitted to the hospital suffering from fatigue, leukocytosis, and hypercalcemia. On admission, laboratory tests revealed that her leukocyte count was elevated to 76x109/L (>80% abnormal lymphocytes). Blood chemistry analysis revealed elevated lactate dehydrogenase (LDH: 808 IU/L) and soluble interleukin-2 receptor (sIL-2R: 43.465 U/mL). Seropositivity for human T-cell leukemia virus type-1 (HTLV-1) was confirmed and monoclonal integration of HTLV-1 was detected by Southern blotting of DNA isolated from peripheral blood (PB). She was diagnosed with acute ATL. Flow cytometric analysis demonstrated that the abnormal lymphocytes were CD3+CD4+CD25+. Computed tomography (CT) revealed multiple lymphadenopathies, involving the supraclavicular fossae and the inguinal, mediastinum, and para-aortic regions.

**Figure 1:**
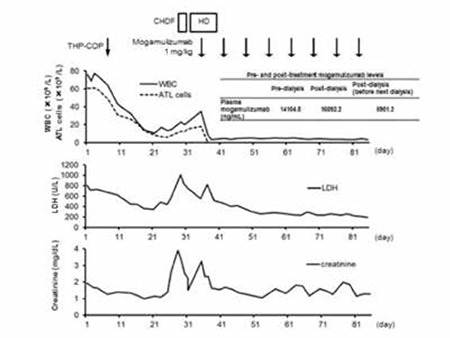
Clinical course: time-points of THP-COP (cyclophosphamide, pirarubicin, vincristine, and prednisolone) and mogamulizumab administration. WBC counts, ATL cell counts, and LDH and creatinine levels throughout the clinical course, and the concentration of mogamulizumab in the plasma pre- and post-dialysis, are shown. CHDF: Continuous hemodiafiltration, HD: hemodialysis.

She was treated with systemic chemotherapy (THP-COP regimen: cyclophosphamide, pirarubicin, vincristine, and prednisolone) on day 8 post-admission. Although the number of ATL cells in the PB gradually decreased, they were persistent. On day 18 of THP-COP, she experienced a high fever and hypotension. Because serum β-D-glucan levels were elevated and a chest CT revealed a pulmonary infiltrate, she was administered cefepime and voriconazole. However, she developed anuric acute renal failure, probably induced by voriconazole, and so emergent hemodialysis was initiated. Subsequently, both the number of ATL cells and her LDH levels increased, suggesting that the THP-COP regimen was not working. Because the ATL cells expressed high levels of CCR4, she received an intravenous infusion of mogamulizumab (1.0 mg/kg) once a week for 8 weeks. The concentration of mogamulizumab in the plasma was measured using an enzyme-linked immunosorbent assay [2,3]. The plasma concentrations of mogamulizumab before and after hemodialysis during the first mogamulizumab infusion and just before the second infusion (Ctrough) were 14,104.8 ng/mL, 16,092.2 ng/mL, and 5901.2 ng/mL, respectively. The plasma levels of mogamulizumab before and after hemodialysis were comparable, and Ctrough was in the range of published data [2], suggesting that therapeutic levels of mogamulizumab may be maintained in patients undergoing dialysis (Figure 1). Five months after the mogamulizumab treatment the leukemia relapsed. However, the patient did not accept any further treatment or a rechallenge with mogamulizumab. She was managed with best supportive care, and she died 10 months after diagnosis with ATL.

ATL is an aggressive peripheral T-cell neoplasm that cannot usually be cured by conventional chemotherapy [4,5]. Allogeneic hematopoietic stem cell transplantation is now considered the only curable treatment for young patients with ATL, but it is not applied to elderly patients because of higher toxicities. Currently, chemotherapy is the only therapeutic option for elderly ATL patients but there are concers about their safety and efficacy [6]. Mogamulizumab is a humanized anti-CCR4 antibody with a defucosylated Fc region and has been used to treat relapsed/refractory CCR4-positive ATL with 50% efficacy in a phase II study [3,7]. The fucose content in the oligosaccharide structure in the Fc region of mogamulizumab is reduced using Potelligent technology, which enhances the antibody-dependent cellular toxicity because of increased binding affinity to the Fcγ receptor on effector cells [8]. More recently, mogamulizumab was also approved for the treatment of relapsed CCR4-positive peripheral T-cell lymphoma and cutaneous T-cell lymphoma in Japan [9].

To date, limited data exist about the application of mogamulizumab in patients undergoing hemodialysis. Here, mogamulizumab treatment resulted in complete remission of an elderly ATL patient with no major adverse events such as infusion reaction or skin rash (including re-exacerbation of renal function). Thus, administration of mogamulizumab may be considered as a safe therapeutic option in this setting. The present case shows that therapeutic mogamulizumab levels can be achieved and maintained in patients undergoing hemodialysis as mogamulizumab is not eliminated by the procedure.

**Conflict of Interest Statement**

The authors of this paper have no conflicts of interest, including specific financial interests, relationships, and/or affiliations relevant to the subject matter or materials included.
